# Comparative studies of deep learning segmentation models for left ventricle segmentation

**DOI:** 10.3389/fpubh.2022.981019

**Published:** 2022-08-25

**Authors:** Muhammad Ali Shoaib, Khin Wee Lai, Joon Huang Chuah, Yan Chai Hum, Raza Ali, Samiappan Dhanalakshmi, Huanhuan Wang, Xiang Wu

**Affiliations:** ^1^Department of Electrical Engineering, Faculty of Engineering, Universiti Malaya, Kuala Lumpur, Malaysia; ^2^Faculty of Information and Communication Technology, BUITEMS, Quetta, Pakistan; ^3^Department of Biomedical Engineering, Faculty of Engineering, Universiti Malaya, Kuala Lumpur, Malaysia; ^4^Department of Mechatronics and Biomedical Engineering, Lee Kong Chian Faculty of Engineering and Science, Universiti Tunku Abdul Rahman, Kajang, Malaysia; ^5^Department of Electronics and Communication Engineering, Faculty of Engineering and Technology, SRM Institute of Science and Technology, Kattankulathur, India; ^6^Institute of Medical Information Security, Xuzhou Medical University, Xuzhou, China

**Keywords:** Convolutional Neural Network (CNN), segmentation, image processing, deep learning, left ventricle (LV), echocardiography

## Abstract

One of the primary factors contributing to death across all age groups is cardiovascular disease. In the analysis of heart function, analyzing the left ventricle (LV) from 2D echocardiographic images is a common medical procedure for heart patients. Consistent and accurate segmentation of the LV exerts significant impact on the understanding of the normal anatomy of the heart, as well as the ability to distinguish the aberrant or diseased structure of the heart. Therefore, LV segmentation is an important and critical task in medical practice, and automated LV segmentation is a pressing need. The deep learning models have been utilized in research for automatic LV segmentation. In this work, three cutting-edge convolutional neural network architectures (SegNet, Fully Convolutional Network, and Mask R-CNN) are designed and implemented to segment the LV. In addition, an echocardiography image dataset is generated, and the amount of training data is gradually increased to measure segmentation performance using evaluation metrics. The pixel's accuracy, precision, recall, specificity, Jaccard index, and dice similarity coefficients are applied to evaluate the three models. The Mask R-CNN model outperformed the other two models in these evaluation metrics. As a result, the Mask R-CNN model is used in this study to examine the effect of training data. For 4,000 images, the network achieved 92.21% DSC value, 85.55% Jaccard index, 98.76% mean accuracy, 96.81% recall, 93.15% precision, and 96.58% specificity value. Relatively, the Mask R-CNN outperformed other architectures, and the performance achieves stability when the model is trained using more than 4,000 training images.

## Introduction

Cardiovascular diseases (CVDs) are the leading cause of death throughout the world (1). Every third person is affected by CVDs each year, according to World Health Organization (WHO) statistics (2, 3). The gold standard in diagnostic imaging of the heart is echocardiography and cardiac magnetic resonance imaging (CMRI). CMRI provides 3-dimensional visualization of the heart with a complete analysis of cardiac structure, whereas echocardiography can examine the function and structure of the heart (4).

However, when compared to echocardiography, CMRI has several disadvantages, including limited availability, time consumption, and cost (5). Echocardiography, on the other hand, is a non-invasive, low-cost, and non-ionization radiation technique, making it the most important and accessible imaging modality for cardiac analysis (6). The assessment of the left ventricle (LV) structures and functions is an important study in cardiac analysis (7). Estimating LV size is necessary for calculating ventricular volume, ejection fraction, wall motion irregularities, and myocardial thickness (8). It identifies high-risk patients and predicts future cardiovascular events (9). As a result, the proper detection of the LV boundary depends on the LV segmentation, which also makes the analysis and diagnosis simple. On the other hand, manual LV detection generally takes a long time, is labor-intensive, and heavily relies on the experience of cardiologists (10). The structural characteristics of ventricles make it a difficult undertaking to segment them. Compared to segmenting organs like the liver or kidney, it is more challenging (11–13). In order to be quicker, more accurate, and less operator-dependent, automatic LV segmentation from echocardiography is required.

Deep learning is widely employed in automatic image segmentation and also in medical image processing. Due to its superior performance, deep learning has emerged as the technology that receives the most attention. Taking into account the significance of deep learning, this article aims to make a comparative analysis of three well known deep learning segmentation architectures, SegNet, FCN, and MaskR-CNN, for LV segmentation. The results obtained by comparing the performance of these algorithms on the same dataset can help to understand these models and to determine which method of image segmentation is most effective for LV segmentation.

## Related work

Various LV segmentation methods, including deformable models, statistical models (14), and machine learning approaches, have been successfully used in recent years. Due to the flexibility in shape representation, deformable models are the most used method for segmentation in ultrasound pictures (15–17). To achieve the intended shape, it employs predetermined curves or surfaces that change the shape under the influence of internal forces. Additionally, these deformable models are used to create LV segmentation (18, 19). The performance of deformable models is enhanced using a variety of transformation techniques. A pSnake approach based on 1-D Hilbert transformation was proposed by Alexandria et al. (20). For reliable and efficient segmentation, S. Zhang presented a deformable framework (21). The model can handle significant faults and reserve shape details. The segmentation of the four main views, including the left ventricle (LV), using 2D echocardiography also uses deformable models (22). A few new parameters were included in the energy function to further enhance the performance of deformable models and achieve high accuracy (23). A new energy function for segmenting the whole myocardium in various directions was defined in the enhanced version of (22). The deformable models are comprehensive and appropriate for segmenting LV, but they have certain restrictions when it comes to choosing the right initialization since most of the methods necessitate human decision-making during the initialization process. These previous models' validity negates the effectiveness and capacity of such strategies. In such circumstances, the parameters must be reparametrized robustly to recover the boundary accurately.

The statistical deformable models are generative models that, through optimization during the fitting process, recover the parametric description of the particular object. Active Shape Models (ASM) and Active Appearance Models (AAM) have been the most widely utilized techniques in echocardiography (AAM). The behavior of the model is created during the training phase by manually tracing over segmented data. The geometry and visual representation of the heart anatomy were all included in the final model. A reliable segmentation of cardiac pictures in terms of space and/or time is ensured by this single model (24). Based on AAM, it is suggested to automatically segment the left ventricle (LV) using a 3D echocardiography approach and to assess the shape and texture of model generalization. The suggested model accurately extrapolates the shape model. However, the texture model struggled in situations where AAM matching (25) was constrained. Carminati et al. (26) created a shape model of the LV using the statistical model on echocardiography data, which was then applied to MRI scans. These statistical models needed several beneficial training examples, and from these samples, the model creates the shape of the item. These models need initialization and shape assumptions in addition to a large number of useful annotated photos. The automatic segmentation of LV is further constrained by its appearance.

Recent research has shown that machine learning approaches are useful for LV segmentation because they do not require initialization and do not rely on assumptions about shape and appearance. Methods based on marginal space learning have primarily been considered for accurately locating the myocardial region (27). Similarly, a border fragment model has been successfully used to identify extracted contours (28). The random forest-based machine learning approach has been used as a discriminative classifier to describe the association of each voxel to the myocardium (29) and is a popular machine learning method for LV segmentation (29–31). Deep learning has attracted attention in image segmentation since it is a completely automatic method (32–34). Deep learning techniques, such as Convolutional Neural Network (CNN), have been used to segment natural images with remarkable results. Deformable models are also used in conjunction with CNN to segment the LV from 3D echocardiography to determine the Region of Interest (ROI) (35–37). Deep learning-based architecture “Fully Convolutional Network (FCN)” (38) is used for LV segmentation in CT images, U-Net on MRI images (39, 40), U-Net on 2D echocardiography images (41), and another U-Net-based architecture “Anatomically Constrained Neural Network (ACNN)” (42) is used for 3D images. Leclerc et al. (43, 44) investigated the performance of U-Net and the amount of data required to train the network for LV segmentation using U-Net.

Inspired by the performance of deep learning methods, three end-to-end fully automatic segmentation models are designed to segment the LV. Stated below are the main contributions of this paper:

1. Implemented the state-of-the-art CNN architectures (SegNet, FCN, and Mask R-CNN) used for image segmentation.
2. Analyzed these CNN architectures by assessing the segmentation mask through evaluation metrics.
3. Determined the trade-off between the amount of data and accuracy using Mask R-CNN architecture.
4. Developed a dataset (for a fair comparison) containing the apical four-chamber view of the heart and binary mask of LV, used for training, validation, and testing the network.

Section Introduction introduces the research topic. Section Related work summarizes the literature used for the LV segmentation and the contribution of the article. Section Materials and method describes the dataset employed, the architecture description of CNN models, network training, and the evaluation metrics used to evaluate the performance of these models. Section Discussion presents the results from applying Segnet, FCN, and Mask R-CNN with 1,000 training images, followed by analyzing the performance of Mask R-CNN with increasing training data. The results are discussed in Section Discussion.

## Materials and methods

### Dataset

The dataset used in this study was obtained retrospectively from the National Heart Institute in Kuala Lumpur, Malaysia. It is made up of 6,000 apical four-chamber 2D echocardiography images. These images were collected using protocol number RD5/04/15, which was approved by the National Heart Institute's Research Ethics Board in Kuala Lumpur, Malaysia. An ultrasound system (Philips IE33) with an S5-1 (1.0–3.0 MHz) transducer was used to perform the 2-D echocardiography. Each image is 800x600 pixels in size, with a resolution of 0.3 mm x 0.3 mm and a frame rate of 30–100 Hz. All images were resized to 512 x 512 to remove the extraneous background. One thousand images are used to train all of the models for the comparison of different CNN models. To examine the impact of training data on performance, a dataset with varying numbers of images, 2,000, 3,000, 4,000, and 5,000, is used to train the model. However, the number of test and validation images remains constant (500 test and 500 validation images). Since these test images were not used in the training process, the trained model is tested using unseen data.

### Network architecture

For LV segmentation, three different CNN models are used: FCN (fully convolutional Network), SegNet (encoder-decoder based), and Mask R-CNN. SegNet is intended to be a fast architecture for pixel-by-pixel semantic segmentation. SegNet's decoder upsamples the low-resolution input provided by previous feature maps. To perform non-linear upsampling, the decoder employs pooling indices computed during the max-pooling step of the corresponding encoder. The FCN, on the other hand, confers several advantages over other models, including the ability to process variable image sizes and handle spatial information. Mask R-CNN was chosen for this study, attributed to its capabilities and robustness for general-purpose object segmentation.

#### Fully convolutional network

**Figure 1:**
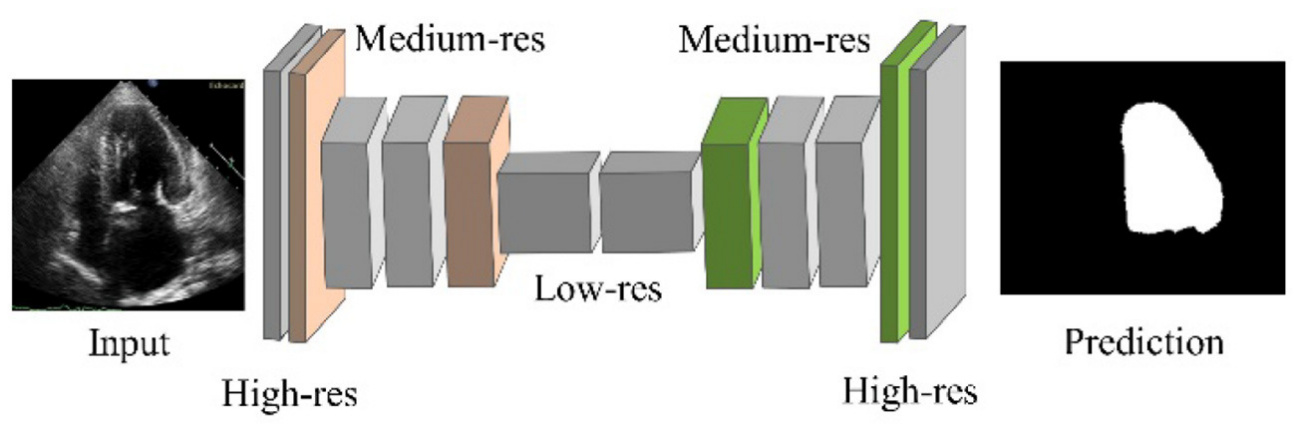
FCN architecture.

For image segmentation, the FCN is a well-known CNN architecture (45). The network is split into two sections: downsampling and upsampling. Downsampling maps complex image features and downsampling spatial resolutions using convolution operations followed by polling. As shown in Figure 1, the output of this step is of very low resolution. Each pixel in semantic segmentation must be classified as LV or background pixel. The un-pooling operation is performed on the pooling operation's low-resolution output to obtain the output mask with the same resolution as the original image. Un-pooling is the process of converting a single value into a collection of new values. The deconvolution process reduces the input image resolutions which impedes the regeneration of finer details. To overcome this caveat, skip connections are adopted to acquire sufficient information to generate the finer segmentation boundaries.

#### SegNet

**Figure 2:**
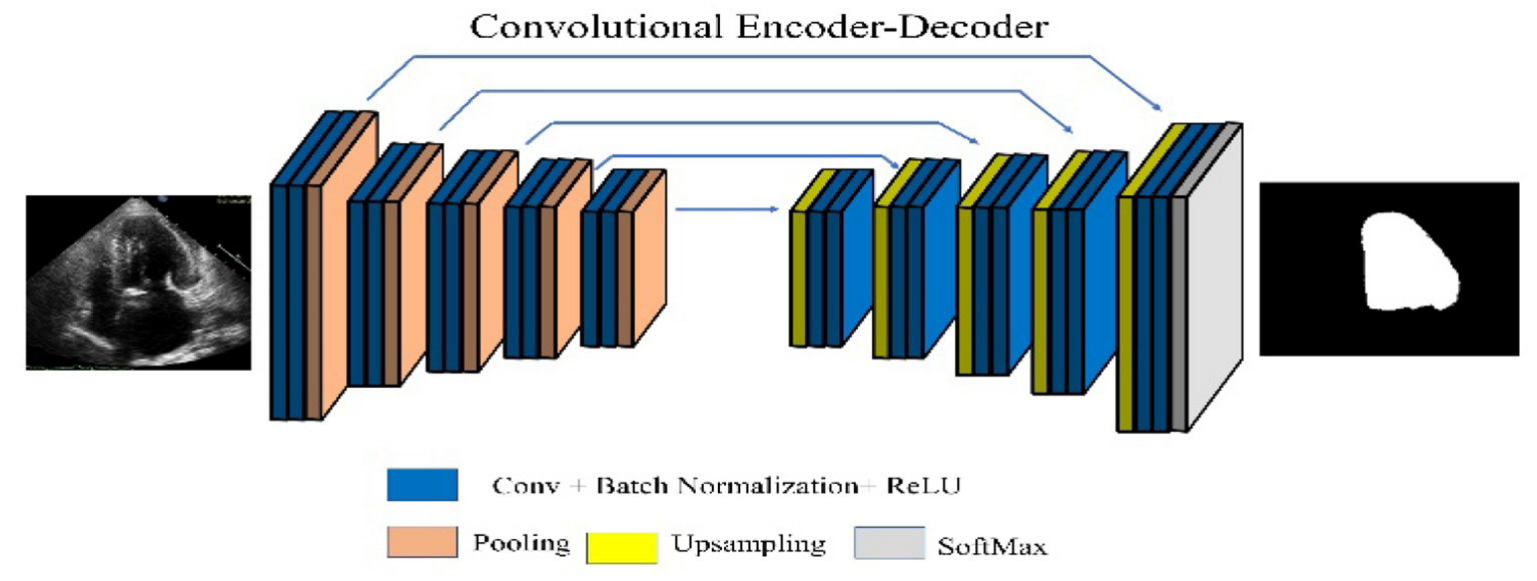
SegNet architecture.

SegNet, one of the popular CNN architectures for segmentation, consists of an encoder and decoder part with a pixel-wise classification layer in the final stage. Object classification is performed by 13 convolutional layers (13-layer VGG network). The output features are passed through batch normalized, followed by ReLU, Max pooling, and stride operations. The decoder layer is designed for each corresponding encoder layer. Hence, the decoder consists of 13 layers. A SoftMax classifier is used at the output of the decoder, yielding the class probabilities of each pixel (35). The SegNet architecture is shown in Figure 2.

#### Mask R-CNN

**Figure 3:**
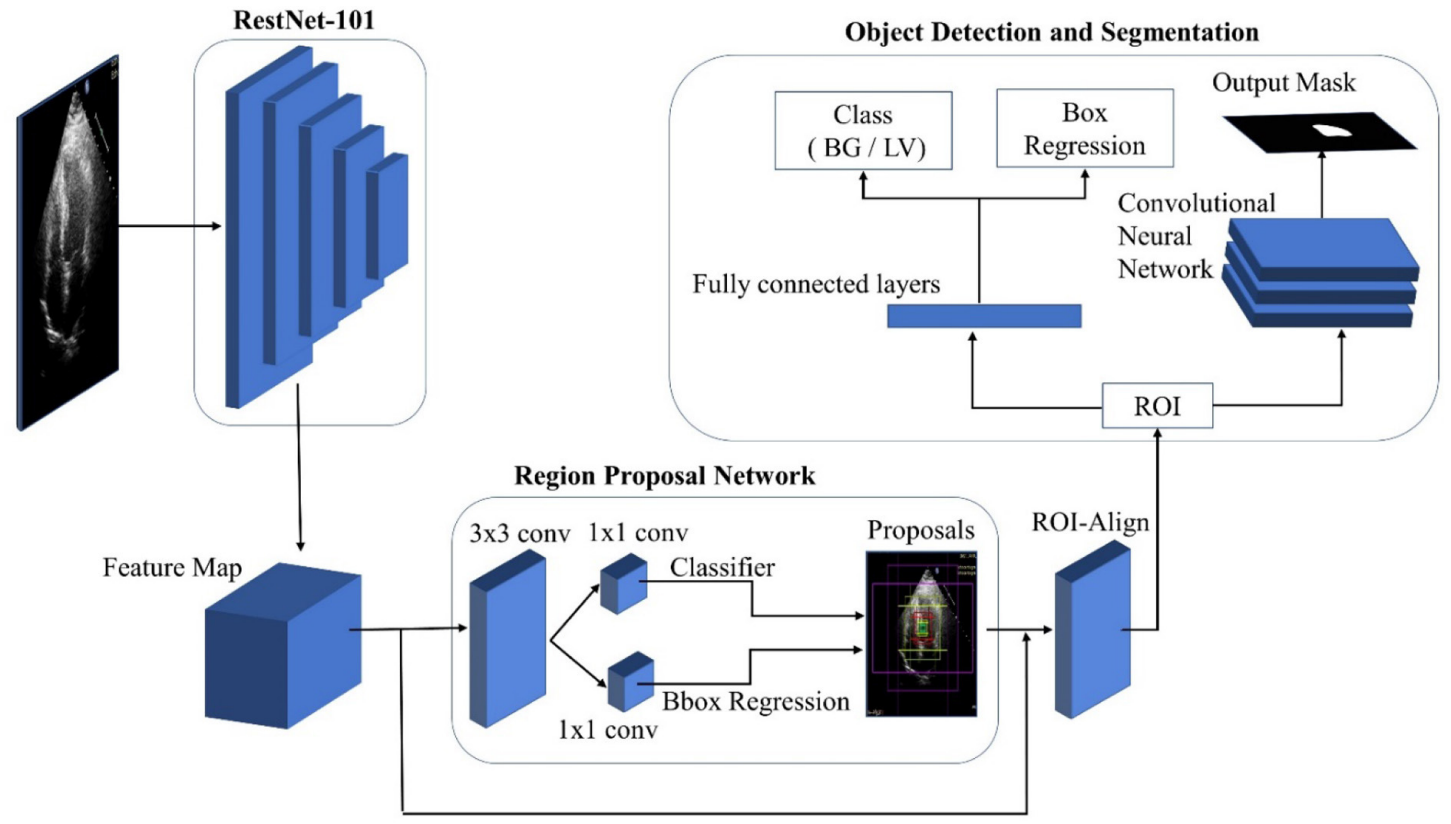
Mask R-CNN architecture.

Figure 3 depicts the general architecture of Mask R-CNN. The ResNet-50-FPN model and the ResNet-101-FPN model are used in the first part of the framework to extract feature maps from images. The backbone network in the model with ResNet-50-FPN consumes less computational load than ResNet-101. With no changes to the model or training procedure, the ResNet-101-FPN achieves higher accuracy. As a result, ResNet-101-FPN is selected as the framework's backbone network. The input image is processed by the backbone CNN architecture to generate the feature map. This feature map serves as the input for the subsequent stages. The Region Proposal Network (RPN) is the framework's second component, and it is used to extract region proposals. The RPN examines the complete image by sliding window technique and proposes the region that might contain the desired object. These regions and the boxes generated by RPN on the image are called anchors, with around two hundred thousand units of different sizes and aspect ratios. In this research, different scales from the original RPN network were used.

Four different scales containing all possible rectangular boxes were defined: 32 × 32, 64 × 64, 128 × 128, and 256 × 256. Three aspect ratios; 0.5, 1.0, and 2.0 are used for a total of 15 proposals for each pixel. The feature map is passed through a 3x3 convolution layer and subsequently two 1 x 1 convolutional layers. The first 1 x 1 convolutional layer classifies the anchor as foreground (LV) or background, and the second convolution layer coordinates the correction of the anchor. A small convolutional network determines the object possibility of each anchor to calculate the anchor score. The top N high score boxes were chosen as ROI based on this score. If multiple anchors overlap, the non-max suppression technique is applied to keep the one with the highest foreground score. The RPN's bounding box refinement steps allow for ROI box size adjustment. Nonetheless, sigmoid classifiers are limited to inputs of a fixed size. As a result, ROI-Align is required, which takes the object proposal and uses bilinear interpolation to compute the feature map values.

The class and ROI mask are processed by separated network heads. In this case, only one ROI is defined, along with foreground and background (FG/BG) classes. With a single ROI, fully connected layers are used to predict the class object. The class of an object is determined by the class-entropy loss function, which calculates class loss. Due to a major single segmentation class, a sigmoid function is used as a classifier (LV).

A convolutional network is used in ROI to generate the mask. Each pixel in ROI is subjected to the sigmoid function, which produces an output mask. Each ground-truth mask and predicted mask were defined as 28 28 during training. This small mask size aids in keeping the mask branch at a low resolution to conserve memory. The predicted masks were scaled up to the size of the original ROI at the output.

### Network training

The network is trained on a workstation equipped with a Core i7 Xeon E5-2620 CPU and 11GB Nvidia GeForce GTX 1080Ti GPU. Different hyperparameters are used to train the model, and finally the network was trained for 50 epochs with 100 training steps per epoch. Initially, the learning rate is set to 0.001 and ends at 0.0001. Stochastic gradient descent is selected as the optimizer with a momentum of 0.9.

### Evaluation metrics

Five hundred images set aside for testing are used to evaluate the trained network. The trained model segments the LV and generates test image segmented binary masks. These segmented binary masks are then compared to the ground truth binary mask of test images. The Dice Similarity Coefficient (DSC) is used to assess model accuracy. The overlap-based method used to calculate the dice similarity index is represented Equation (1) (46). S_GT_ in the equation represents the ground truth image, which includes the original LV size and boundary. The model's segmented mask is represented by the S_Seg_. The DSC is calculated by dividing the intersected regions of two masks by the total region of both masks. DSC has a range of [0 1], with 0 representing no similarity or non-overlapping region and 1 representing exact overlapping.


(1) $$\begin{array}{r} DSC=\;\frac{2|S_{GT}\;\cap \;S_{Seg}|}{\left|S_{GT}|\;+\;|S_{Seg}\right|} \end{array}$$

The Equation (2) denotes the Jaccard index (47) (also known as intersection over union or IoU). The Jaccard index penalizes over- and under-segmentation more than the DSC.


(2) $$\begin{array}{r} Jaccard=\;\frac{S_{GT}\cap \;S_{Seg}}{S_{GT}\cup \;S_{Seg}} \end{array}$$

The segmentation performance of the models is also evaluated using pixel accuracy. It simply calculates the percentage of pixels that are correctly classified. It is typically calculated for each class individually as well as for all classes. Equation (3) is used to calculate the accuracy.


(3) $$\begin{array}{r} Pixel\;Accuracy=\;\frac{TP+TN}{TP+TN+FP+FN} \end{array}$$

where TP is True Positive which represents the correctly classified pixels of the segmented class. The incorrectly classified pixels of the segmented class are FP i.e., False Positive. Similarly, TN is a True negative background pixel correctly classified, and FN is a False Negative indicating the incorrectly classified pixels of the background.

Three other evaluation metrics; recall, precision, and specificity are also adopted to evaluate the segmented mask. Recall, also known as sensitivity or true positive rate, focuses on the true positive detection capabilities. Specificity, also called True Negative Rate (TNR), is the percentage of negative pixels (background) in the ground truth segmentation that are also negative pixels in the segmentation being tested. The recall, precision, and specificity are computed by the formulas presented in Equations (4), (5), and, (6) respectively.


(4) $$\begin{array}{r} Recall=\;\frac{TP}{TP+FN} \end{array}$$

(5) $$\begin{array}{r} Precision=\;\frac{TP}{TP+FP} \end{array}$$

(6) $$\begin{array}{r} Specificity=\;\frac{TN}{TN+FP} \end{array}$$

## Results

The SegNet, FCN, and Mask R-CNN architectures are trained using 1,000 training images and the networks are evaluated using 500 test images. Table 1 shows the average values of DSC, Jaccard index, pixel accuracy, recall, precision, and specificity of the three architectures.

**Table 1:** Minimum, maximum, and mean values with a standard deviation of evaluation metrics for three models.

**CNN Model**	**DSC**			**Jaccard index**			**Accuracy**			**Recall**			**Precision**			**Specificity**		
	**min**	**max**	**Mean ±std**	**min**	**max**	**Mean ±std**	**min**	**max**	**Mean ±std**	**min**	**max**	**Mean ±std**	**min**	**max**	**Mean ±std**	**min**	**max**	**Mean ±std**
SegNet	0.6492	0.8884	0.7651 ± 0.0401	0.4806	0.7992	0.6195 ± 0.0453	0.7524	0.9286	0.8455 ± 0.025	0.3261	0.7981	0.7486 ± 0.301	0.4813	0.8351	0.6519 ± 0.055	0.5824	0.8621	0.6891 ± 0.045
FCN	0.7106	0.9279	0.8386 ± 0.0342	0.5511	0.8654	0.7221 ± 0.0412	0.8627	0.9705	0.9193 ± 0.019	0.4568	0.9872	0.9649 ± 0.174	0.6013	0.8041	0.7238 ± 0.043	0.6871	0.8877	0.7891 ± 0.044
Mask R-CNN	0.7567	0.9958	0.8831 ± 0.0356	0.6086	0.9916	0.7907 ± 0.0401	0.8903	0.9981	0.9457 ± 0.018	0.509	0.9910	0.9681 ± 0.216	0.6321	0.8182	0.7937 ± 0.031	0.7056	0.9021	0.8157 ± 0.033

The SegNet results in the lowest average DSC level (0.7651), Jaccard (0.6195), mean accuracy (0.8455), recall (0.7486), precision (0.6519), and Specificity (0.6891).

**Figure 4:**
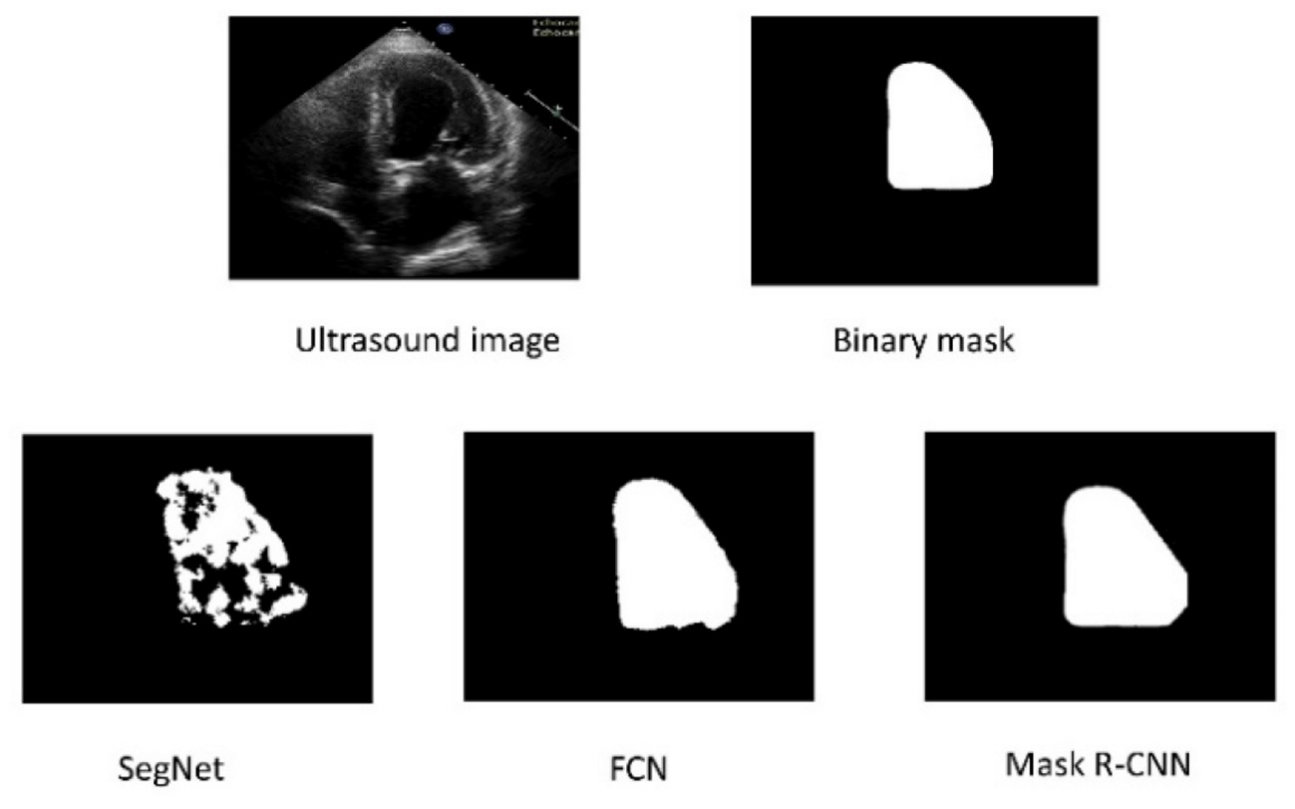
The top row represents the original Ultrasound image and ground truth binary mask. The bottom row shows the segmented results of the three architectures.

Figure 4 depicts these findings pictorially. The second row shows the predicted mask output from SegNet, FCN, and Mask R-CNN, in that order. The SegNet architecture fails to accurately predict labels for both boundaries and within the region. FCN outperforms SegNet in predicting boundaries and inside regions. Compared to SegNet and FCN, the Mask R-CNN achieves better LV boundary and inside region segmentation.

The Mask R-CNN is chosen to investigate the impact of training data size on segmentation performance based on the results of 1,000 training images presented in Table 1 and Figure 4. The Mask R-CNN model is evaluated by gradually increasing the size of the training data. The trained model is evaluated for each dataset by measuring the evaluation metrics on 500 test images.

**Figure 5:**
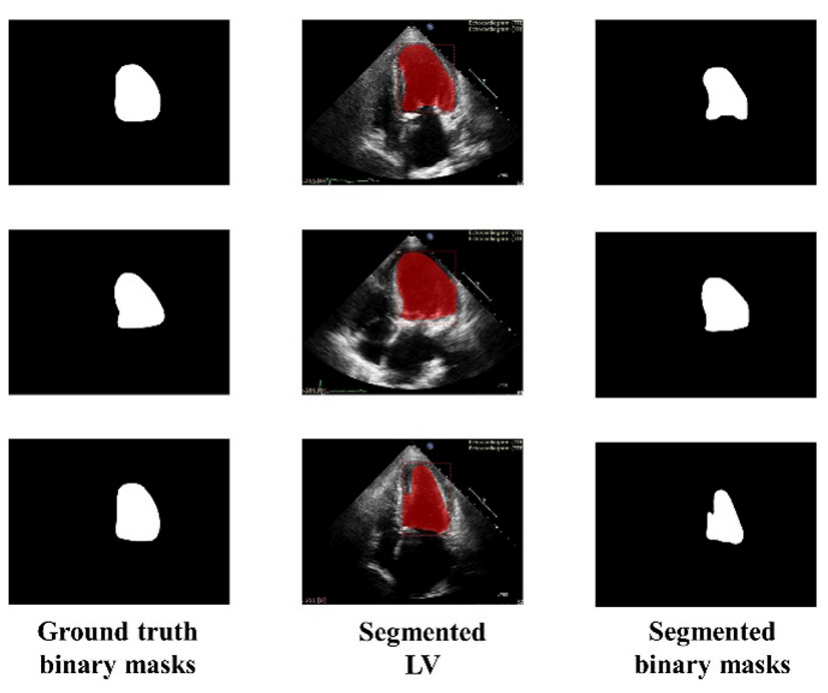
Ground truth binary mask, segmented LV, and corresponding segmented binary masks (Using 2,000 images).

Figure 5 shows the three random samples of the trained model using 2,000 images. Ground truth binary masks of test images are shown in the first column, segmented LV in the second column, and segmented binary mask is presented in the third column.

**Figure 6:**
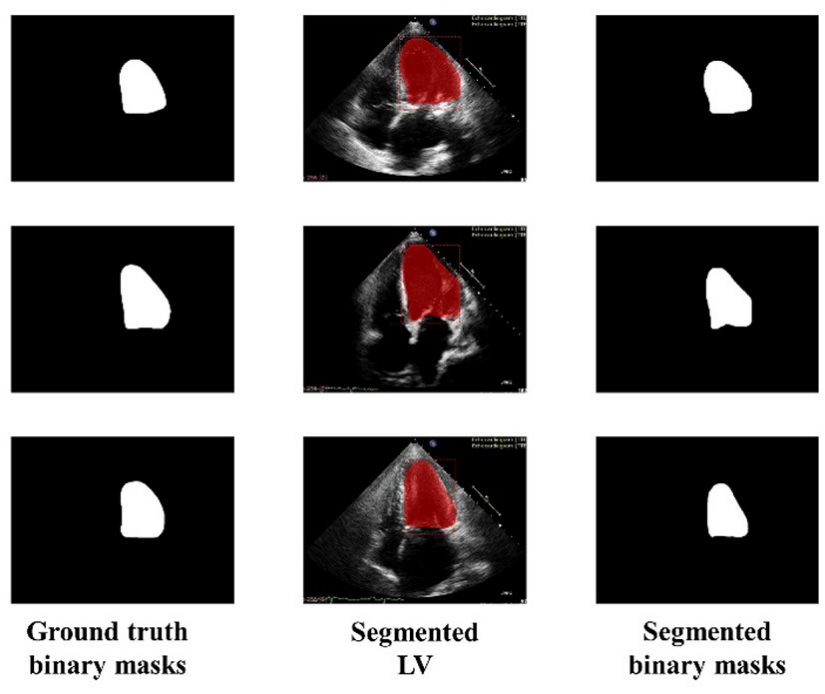
Ground truth binary mask, segmented LV, and corresponding segmented binary masks (Using 3,000 images).

**Figure 7:**
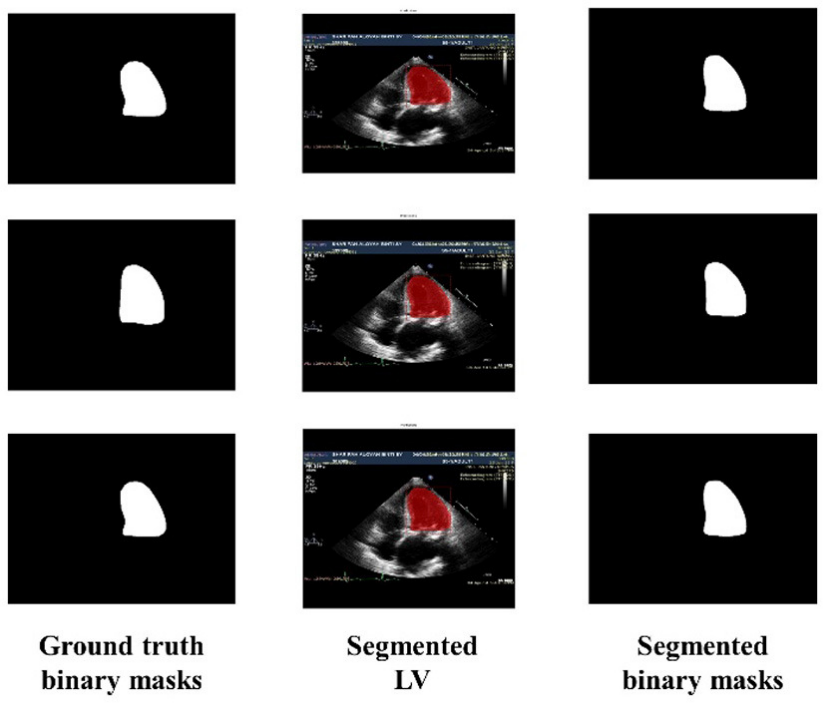
Ground truth binary mask, segmented LV, and corresponding segmented binary masks (using 4,000 images).

Similarly, Figure 6 shows the output of the trained model using 3,000 images. In comparison to Figure 5, the results are improved with an average DSC of 0.8945 and a mean accuracy of 0.9581. Figure 7 shows the results of 4,000 training images. The model was eventually trained by increasing the training data to 5,000 images. The model's performance became saturated, and no significant improvement in evaluation metrics was observed. The obtained average DSC and mean accuracy values are 0.9228 and 0.9881, respectively.

The performance of the Mask R-CNN is evaluated by gradually increasing the training data size. Table 2 shows the model's average values for all six evaluation metrics for different training data sizes. As training data is one of the key parameters for the performance of deep learning, increasing the training data improves the average values of evaluation metrics. The results show that increasing the training data improves Mask R-CNN performance with better LV segmentation.

**Table 2:** Mean with standard deviation values of evaluation metrics using different training data size.

**Training data (Number of images)**	**DSC (Mean ±std)**	**Jaccard index (Mean ±std)**	**Accuracy (Mean ±std)**	**Recall (Mean ±std)**	**Precision (Mean ±std)**	**Specificity (Mean ±std)**
1,000	0.8831 ± 0.0356	0.7907 ± 0.0401	0.9457 ± 0.018	0.9681 ± 0.216	0.7937 ± 0.055	0.8057 ± 0.033
2,000	0.8945 ± 0.0365	0.8091 ± 0.0372	0.9581 ± 0.018	0.9712 ± 0.201	0.8398 ± 0.052	0.8462 ± 0.057
3,000	0.9071 ± 0.0281	0.8299 ± 0.0313	0.9703 ± 0.016	0.9809 ± 0.170	0.8730 ± 0.056	0.899 ± 0.041
4,000	0.9221 ± 0.0237	0.8555 ± 0.0294	0.9876 ± 0.015	0.9902 ± 0.165	0.9315 ± 0.049	0.9658 ± 0.040
5,000	0.9228 ± 0.0233	0.8566 ± 0.2899	0.9881 ± 0.015	0.9903 ± 0.163	0.9317 ± 0.050	0.9660 ± 0.042

## Discussion

In the study of the comparison of three different segmentation models, the SegNet was the only one that was unable to segment the LV accurately and also could not correctly segment the LV inside the boundary, as shown in Figure 4. Due to the small size of its classes and the lack of convolutional architecture, SegNet has achieved a DSC value of 0.7651, a Jaccard index of 0.6195, accuracy of 0.8455, recall 0.7486, precision 0.6519, and specificity of 0.6819. The result reflects the inability of SegNet in capturing the global context of objects. The FCN achieves superior LV segmentation within the area, resulting in DSC value of 0.8386, 0.7221 of Jaccard index, 0.9193 accuracy, 0.9646 recall, 0.7328 precision, and specificity 0.7891, which are considerably better than SegNet.

FCN extracts the features through downsampling and then restores the image features through upsampling. This technique improves the extraction of features. However, this sequence of downsampling and upsampling might compromise segmentation owing to the loss of image detail. Mask R-CNN has shown the ability to effectively segment LV due to its architecture (which first suggests the region containing the LV followed by applying the ROI Align module for precise localization, and then finally separating the LV from the ROI as the subsequent step by a convolutional network). This process improves the accuracy of segmentation.

**Figure 8:**
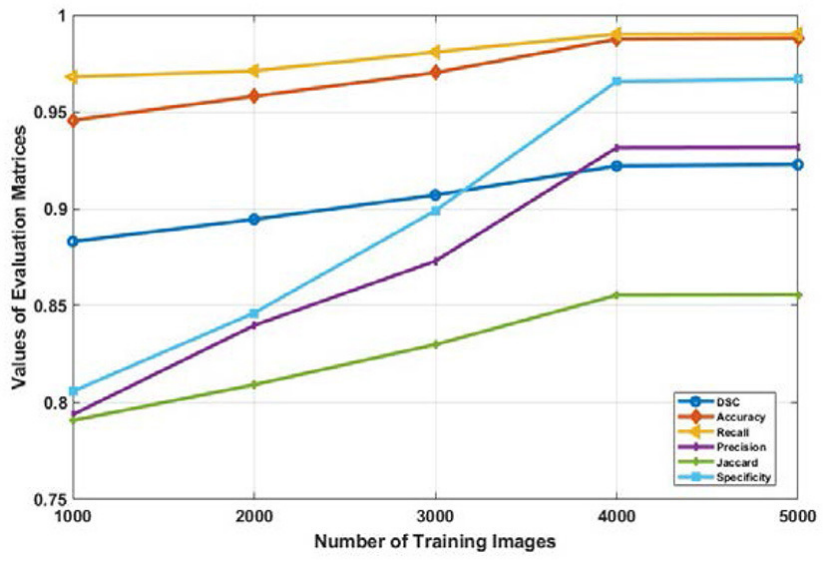
Average values of evaluation metrics for different data size used for training the Mask R-CNN model.

The impact of the size of the training data set on the overall segmentation performance of the model was also investigated in this research. The Mask R-CNN model is used for this analysis since among the three models, it exhibited the highest values across all evaluation metrics. It is observable that there is a significant performance improvement when the data size is gradually raised from 1000 to 4000 images, as illustrated in the Figure 8.

The values DSC, Jaccard, accuracy, sensitivity, recall, and specificity increases from 0.8831, 0.7907, 0.9457, 0.9681, 0.7937, 0.333, and 0.8157 to 0.9221, 0.8555, 0.9876, 0.9902, 0.9315, 0.9558 respectively. But the performance of the model trained with 4000 or more input images became consistent. This is depicted in Figure 8 as the graph of all evaluation metrics is almost horizontal when data is increased from 4,000 to 5,000 images. It is evident from these results that the amount of training data has a significant effect on the performance of the model. However, after a certain number of training data sets, the performance of the deep learning model becomes constant.

## Conclusion

One of the most important aspects of diagnosing cardiac disease is LV segmentation. Precise segmentation of the LV impacts significantly on our understanding toward the normal anatomy of the heart, as well as our ability to distinguish the aberrant or diseased structure of the heart. This article compares the SegNet, FCN, and Mask R-CNN architectures in segmenting the LV from echocardiography images. All networks were trained using a self-collected apical four chamber view of the echocardiography dataset. After training the models on 1,000 images, the performance of three architectures is compared. The experimental results showed that the Mask R-CNN architecture surpassed the SegNet and FCN architectures with a DSC of 0.8831, Jaccard index of 0.7907, accuracy of 0.9457, a recall of 0.9681, precision of 0.7937, and specificity of 0.8157. This work also takes into account the influence of training data size on segmentation performance. Due to its superior performance among the three models, the Mask R-CNN model was chosen for analysis. Mask R-CNN was trained using 2,000, 3,000, 4,000, and 5,000 images, and its performance improved as the training data size increased. After 4,000 echocardiography images, the model's performance was saturated, and no significant changes were observed thereafter. The evaluation metrics values on the dataset consisting of 5,000 images did not demonstrate a significant improvement. In future work, this work will be extended to segment the endocardium and epicardium of the LV, aiming to contribute to measuring the end-diastolic volume, end-systolic volume, and ejection fraction.

## Data availability statement

The original contributions presented in the study are included in the article/supplementary material, further inquiries can be directed to the corresponding authors.

## Ethics statement

All the images are collected using protocol number RD5/04/15 approved by the Research Ethics Board of National Heart Institute, Kuala Lumpur, Malaysia.

## Author contributions

All authors listed have made a substantial, direct, and intellectual contribution to the work and approved it for publication.

## Funding

This work was supported in part by Fundamental Research Grant Scheme (FRGS), Ministry of Higher Education Malaysia, and Universiti Malaya under the Grant Number FRGS/1/2019/TK04/UM/01/2.

## Conflict of interest

The authors declare that the research was conducted in the absence of any commercial or financial relationships that could be construed as a potential conflict of interest.

## Publisher's note

All claims expressed in this article are solely those of the authors and do not necessarily represent those of their affiliated organizations, or those of the publisher, the editors and the reviewers. Any product that may be evaluated in this article, or claim that may be made by its manufacturer, is not guaranteed or endorsed by the publisher.

## References

[1] Roth GA Johnson C Abajobir A Abd-Allah F Abera SF Abyu G. Global, regional, and national burden of cardiovascular diseases for 10 causes, 1990 to 2015. J Am Coll Cardiol. (2017) 70:1–25. 10.1016/j.jacc.2017.04.052 28527533 PMC5491406

[2] Laslett LJ Alagona P Clark BA Drozda JP Saldivar F Wilson SR. The worldwide environment of cardiovascular disease : prevalence, diagnosis, therapy, and policy issues a report from the American college of cardiology. J Am Coll Cardiol. (2012) 60:S1–49. 10.1016/j.jacc.2012.11.002 23257320

[3] Cardiovascular Diseases (CVDs). Available online at: https://www.who.int/news-room/fact-sheets/detail/cardiovascular-diseases-(cvds) (accessed May 18, 2020).

[4] Liew YM McLaughlin RA Chan BT Abdul Aziz YF Chee KH Ung NM. Motion corrected LV quantification based on 3D modelling for improved functional assessment in cardiac MRI. Phys Med Biol. (2015) 60:2715–33. 10.1088/0031-9155/60/7/2715 25768708

[5] Irshad M Sharif M Yasmin M Khan A. A survey on left ventricle segmentation techniques in cardiac short axis MRI. Curr Med Imaging Rev. (2018) 14:223–37. 10.2174/1573405613666170117124934

[6] Alfakih K Reid S Jones T Sivananthan M. Assessment of ventricular function and mass by cardiac magnetic resonance imaging. Eur Radiol. (2004) 14:1813–22. 10.1007/s00330-004-2387-0 15258823

[7] Khalil A Ng SC Liew YM Lai KW. An overview on image registration techniques for cardiac diagnosis and treatment. Cardiol Res Pract. (2018) 2018:1437125. 10.1155/2018/1437125 30159169 PMC6109558

[8] Huang X Dione DP Compas CB Papademetris X Lin BA Bregasi A. Contour tracking in echocardiographic sequences via sparse representation and dictionary learning. Med Image Anal. (2014) 18:253–71. 10.1016/j.media.2013.10.012 24292554 PMC3946038

[9] Anderson JL. 2013 ACCF/AHA guideline for the management of ST-elevation myocardial infarction: executive summary: a report of the American college of cardiology foundation/American heart association task force on practice guidelines. Circulation. (2013) 127:529–55. 10.1161/CIR.0b013e3182742c84 23247303

[10] Sonka M Liang W Kanani P Allan J DeJong S Kerber R. Intracardiac echocardiography: computerized detection of left ventricular borders. Int J Card Imaging. (1998) 14:397–411. 10.1023/A:1006114907352 10453395

[11] Goceri N Goceri E. A Neural Network Based Kidney Segmentation from MR Images. In: 2015 IEEE 14th International Conference on Machine Learning and Applications (ICMLA). Miami, FL: IEEE (2015). p. 1195–8. 10.1109/ICMLA.2015.229

[12] Goceri E. A Comparative Evaluation for Liver Segmentation From Spir Images and a Novel Level Set Method Using Signed Pressure Force Function (2013).

[13] Goceri E Unlu MZ Guzelis C Dicle O. An automatic level set based liver segmentation from MRI data sets. In: 2012 3rd International Conference on Image Processing Theory, Tools and Applications (IPTA). Istanbul: IEEE (2012). p. 192–7. 10.1109/IPTA.2012.6469551

[14] Dziri H Cherni MA Ben-Sellem D. New hybrid method for left ventricular ejection fraction assessment from radionuclide ventriculography images. Curr Med Imaging Former Curr Med Imaging Rev. (2021) 17:623–33. 10.2174/1573405616666201118122509 33213328

[15] Spencer KT Bednarz J Mor-Avi V DeCara J Lang RM. Automated endocardial border detection and evaluation of left ventricular function from contrast-enhanced images using modified acoustic quantification. J Am Soc Echocardiogr. (2002) 15:777–81. 10.1067/mje.2002.120505 12174346

[16] Katouzian A Angelini ED Carlier SG Suri JS Navab N Laine AF. state-of-the-art review on segmentation algorithms in intravascular ultrasound (IVUS) images. IEEE Trans Inf Technol Biomed. (2012) 16:823–34. 10.1109/TITB.2012.2189408 22389156

[17] McInerney T Terzopoulos D. Deformable models in medical image analysis: a survey. Med Image Anal. (1996) 1:91–108. 10.1016/S1361-8415(96)80007-7 9873923

[18] Zhu Y Papademetris X Sinusas AJ Duncan JS. A coupled deformable model for tracking myocardial borders from real-time echocardiography using an incompressibility constraint. Med Image Anal. (2010) 14:429–48. 10.1016/j.media.2010.02.005 20350833 PMC4318707

[19] Jahanzad Z Liew YM Bilgen M McLaughlin RA Leong CO Chee KH. Regional assessment of LV wall in infarcted heart using tagged MRI and cardiac modelling. Phys Med Biol. (2015) 60:4015–31. 10.1088/0031-9155/60/10/4015 25919317

[20] de Alexandria AR Cortez PC Bessa JA de Alexandria AR de Abreu JS da Silva Félix JH. pSnakes: a new radial active contour model and its application in the segmentation of the left ventricle from echocardiographic images. Comput Methods Programs Biomed. (2014) 116:260–73. 10.1016/j.cmpb.2014.05.009 24957548

[21] Zhang S Zhan Y Metaxas DN. Deformable segmentation via sparse representation and dictionary learning. Med Image Anal. (2012) 16:1385–96. 10.1016/j.media.2012.07.007 22959839

[22] Dietenbeck T Alessandrini M Barbosa D D'hooge J Friboulet D Bernard O. Detection of the whole myocardium in 2D-echocardiography for multiple orientations using a geometrically constrained level-set. Med Image Anal. (2012) 16:386–401. 10.1016/j.media.2011.10.003 22119489

[23] Dietenbeck T Barbosa D Alessandrini M Jasaityte R Robesyn V. D'hooge J, et al. Whole myocardium tracking in 2D-echocardiography in multiple orientations using a motion constrained level-set. Med Image Anal. (2014) 18:500–14. 10.1016/j.media.2014.01.005 24561989

[24] Mitchell SC Bosch JG Lelieveldt BPF Van der Geest RJ Reiber JHC Sonka M. 3-D active appearance models: segmentation of cardiac MR and ultrasound images. IEEE Trans Med Imaging. (2002) 21:1167–78. 10.1109/TMI.2002.804425 12564884

[25] Van Stralen M Leung KYE Voormolen MM De Jong N Van Der Steen AFW Reiber JHC. Automatic segmentation of the left ventricle in 3D echocardiography using active appearance models. In: Proceedings of IEEE Ultrasonics Symposium. New York, NY: IEEE (2007). p. 1480–3. 10.1109/ULTSYM.2007.372

[26] Carminati MC Piazzese C Pepi M Tamborini G Gripari P Pontone G. A statistical shape model of the left ventricle from real-time 3D echocardiography and its application to myocardial segmentation of cardiac magnetic resonance images. Comput Biol Med. (2018) 96:241–51. 10.1016/j.compbiomed.2018.03.013 29653353

[27] Yang L Georgescu B Zheng Y Wang Y Meer P Comaniciu D. Prediction based collaborative trackers (PCT): a robust and accurate approach toward 3d medical object tracking. IEEE Trans Med Imaging. (2011) 30:1921–32. 10.1109/TMI.2011.2158440 21642040

[28] Stebbing R V Noble JA. Delineating anatomical boundaries using the boundary fragment model. Med Image Anal. (2013) 17:1123–36. 10.1016/j.media.2013.07.001 23941869

[29] Lempitsky V Verhoek M Noble JA Blake A. Random forest classification for automatic delineation of myocardium in real-time 3D echocardiography. Comput Sci. (2009) 5528:447–56. 10.1007/978-3-642-01932-6_48

[30] Milletari F Yigitsoy M Navab N. Left ventricle segmentation in cardiac ultrasound using hough-forests with implicit shape and appearance priors. Midas J. (2014) 49–56. 10.54294/y9qm6j

[31] Domingos JS Stebbing R V Leeson P Noble JA. Structured Random Forests for Myocardium Delineation in 3D Echocardiography. (2014). p. 215–22. 10.1007/978-3-319-10581-9_27

[32] Keraudren K Oktay O Shi W Hajnal J V Rueckert D. Endocardial 3D ultrasound segmentation using autocontext random forests. Midas J. (2014) 41–8. 10.54294/wu2mi1

[33] Chen L-C Papandreou G Kokkinos I Murphy K Yuille AL. DeepLab: semantic image segmentation with deep convolutional nets, atrous convolution, and fully connected CRFs. IEEE Trans Pattern Anal Mach Intell. (2018) 40:834–48. 10.1109/TPAMI.2017.2699184 28463186

[34] Milletari F Navab N Ahmadi SA. V-Net: Fully convolutional neural networks for volumetric medical image segmentation. In: Proceedings of 2016 4th International Conference 3D Vision, 3DV 2016. Standford, CA (2016). p. 565–71. 10.1109/3DV.2016.79

[35] Badrinarayanan V Kendall A Cipolla R. SegNet: A deep convolutional encoder-decoder architecture for image segmentation. IEEE Trans Pattern Anal Mach Intell. (2017) 39:2481–95. 10.1109/TPAMI.2016.2644615 28060704

[36] Dong S Luo G Sun G Wang K Zhang H. A Left Ventricular Segmentation Method on 3D Echocardiography using Deep Learning and Snake. (2016). p. 473–6. 10.22489/CinC.2016.136-409

[37] Dong S Luo G Wang K Cao S Li Q Zhang H. A combined fully convolutional networks and deformable model for automatic left ventricle segmentation based on 3D echocardiography. Biomed Res Int. (2018) 2018:5682365. 10.1155/2018/5682365 30276211 PMC6151364

[38] Koo HJ Lee JG Ko JY Lee G Kang JW Kim YH. Automated segmentation of left ventricular myocardium on cardiac computed tomography using deep learning. Korean J Radiol. (2020) 21:660–9. 10.3348/kjr.2019.0378 32410405 PMC7231613

[39] Zabihollahy F Rajchl M White JA Ukwatta E. Fully automated segmentation of left ventricular scar from 3D late gadolinium enhancement magnetic resonance imaging using a cascaded multi-planar U-Net (CMPU-Net). Med Phys. (2020) 47:1645–55. 10.1002/mp.14022 31955415

[40] Yang X Tjio G Yang F DIng J Kumar S Leng S. A multi-channel deep learning approach for segmentation of the left ventricular endocardium from cardiac images. Proc Annu Int Conf IEEE Eng Med Biol Soc. (2019) 2019:4016–9. 10.1109/EMBC.2019.8856833 31946752

[41] Smistad E Ostvik A Haugen BO Lovstakken L. 2D left ventricle segmentation using deep learning. In: IEEE International Ultrasonic Symposium. Washington, DC: IEEE (2017). p. 4–7. 10.1109/ULTSYM.2017.8092812

[42] Oktay O Ferrante E Kamnitsas K Heinrich M Bai W Caballero J. Anatomically constrained neural networks (ACNNs): application to cardiac image enhancement and segmentation. IEEE Trans Med Imaging. (2018) 37:384–95. 10.1109/TMI.2017.2743464 28961105

[43] Leclerc S Smistad E Grenier T Lartizien C Ostvik A Espinosa F. Deep learning applied to multi-structure segmentation in 2d echocardiography: a preliminary investigation of the required database size. IEEE Int Ultrason Symp. (2018) 2018:1–4. 10.1109/ULTSYM.2018.8580136

[44] Leclerc S Smistad E Pedrosa J Ostvik A Cervenansky F Espinosa F. Deep learning for segmentation using an open large-scale dataset in 2D echocardiography. IEEE Trans Med Imaging. (2019) 38:2198–210. 10.1109/TMI.2019.2900516 30802851

[45] Shelhamer E Long J Darrell T. Fully convolutional networks for semantic segmentation. IEEE Trans Pattern Anal Mach Intell. (2017) 39:640–51. 10.1109/TPAMI.2016.2572683 27244717

[46] Khalil A Faisal A Lai KW Ng SC Liew YM. 2D to 3D fusion of echocardiography and cardiac CT for TAVR and TAVI image guidance. Med Biol Eng Comput. (2017) 55:1317–26. 10.1007/s11517-016-1594-6 27830464

[47] Taha A Hanbury A. Metrics for evaluating 3D medical image segmentation: Analysis, selection, and tool. BMC Med Imaging. (2015) 15:29. 10.1186/s12880-015-0068-x 26263899 PMC4533825

